# Conservative Management of Major Liver Necrosis after Angioembolization in a Patient with Blunt Trauma

**DOI:** 10.1155/2013/954050

**Published:** 2013-12-29

**Authors:** Husham Abdelrahman, Ahmad Ajaj, Sajid Atique, Ayman El-Menyar, Hassan Al-Thani

**Affiliations:** ^1^Trauma Surgery Section, Hamad General Hospital, HMC, P.O. Box 3050, Doha, Qatar; ^2^Clinical Research, Trauma Surgery Section, Hamad General Hospital, Doha, Qatar; ^3^Clinical Medicine, Weill Cornell Medical College, Doha, Qatar

## Abstract

Management of liver injury is challenging particularly for the advanced grades. Increased utility of nonoperative management strategies increases the risk of developing massive liver necrosis (MLN). We reported a case of a 19-year-old male who presented with a history of motor vehicle crash. Abdominal computerized tomography (CT) scan revealed large liver laceration (Grade 4) with blush and moderate free hemoperitoneum in 3 quadrants. Patient was managed nonoperatively by angioembolization. Two anomalies in hepatic arteries origin were reported and both vessels were selectively cannulated and bilateral gel foam embolization was achieved successfully. The patient developed MLN which was successfully treated conservatively. The follow-up CT showed progressive resolution of necrotic areas with fluid replacement and showed remarkable regeneration of liver tissues. We assume that patients with high-grade liver injuries could be managed successfully with a carefully designed protocol. Special attention should be given to the potential major associated complications. A tailored multidisciplinary approach to manage the subsequent complications would represent the best recommended strategy for favorable outcomes.

## 1. Introduction

Trauma is a major contributor to death in the first four decades of life [1]. Abdomen represents the third most commonly injured part of the body following multiple trauma [2]. In particular, liver is the most frequently injured solid organ followed by the spleen in blunt abdominal injuries [3, 4]. Moreover, the recognized high association of mortality with liver injuries had urged the trauma surgeons to look for alternative interventions aiming at a better outcome [4]. The surgical options for liver injury are relatively limited as liver surgery is more challenging for the average experienced surgeons and is often associated with high mortality [5–7]. The significance of nonoperative management of blunt hepatic trauma is well documented and is currently considered as the gold standard care for hemodynamically stable patients [6, 8–10].

Transarterial embolization (TAE) has been introduced as a useful and effective tool for the management of hemodynamically stable patients with blunt hepatic trauma in the multimodality approach [7]. The wide implication of this modality has brought along a group of unique complications such as massive liver necrosis (MLN) or major devascularizing injury which is of a particular clinical importance [11]. However, emergent laparotomy remains the recommended approach for the patient who becomes hypotensive during computerized tomography (CT) scanning or angiography despite fluid resuscitation [12, 13]. Herein, we report a case of severe blunt abdominal trauma with grade four liver injuries which was treated nonoperatively with the aid of TAE.

## 2. Case Report

A 19-year-old male presented with a history of motor vehicle crash. The unrestrained driver sustained mild head and chest injuries (right first rib fracture and occult pneumothorax). Upon physical examination, he was fully conscious with a Glasgow Coma Scale (GCS) of 15 and normotensive but tachycardic. Abdominal examination revealed mild generalized distension and diffuse tenderness mainly at the right upper quadrant with normal bowel sounds, digital rectal examination, and clear urine.

**Figure 1:**
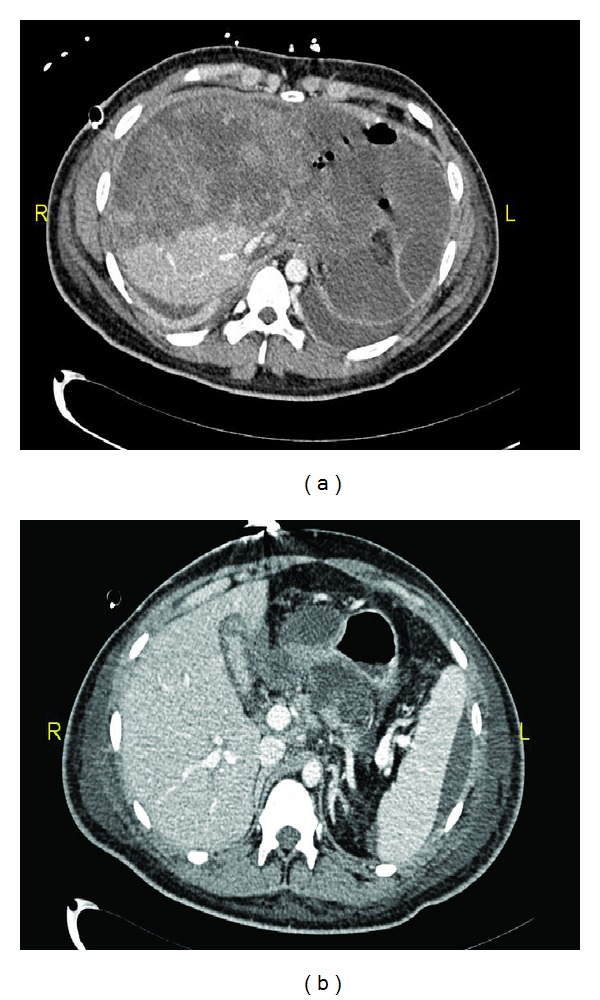
(a) Admission IV contrasts abdomen CT scan showing extensive liver injury with active blush; (b) fluid around spleen and liver.

**Figure 2:**
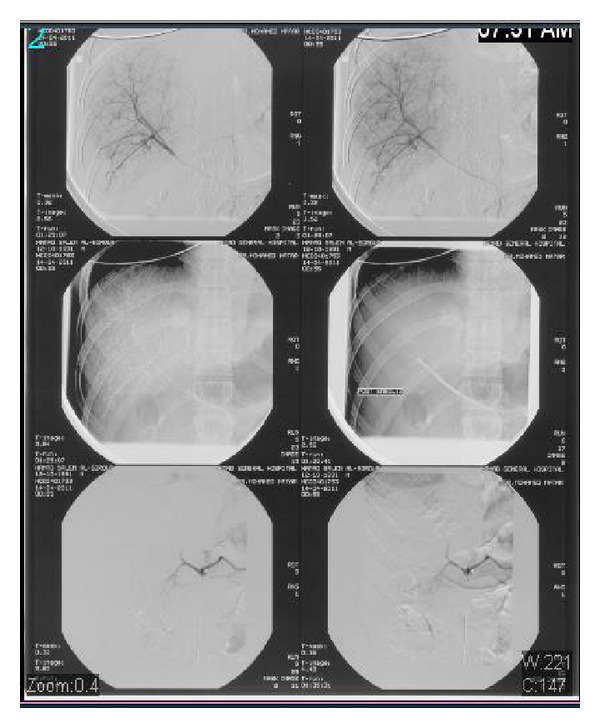
Selective angiogram of hepatic vessels with evidence of active blush, foam embolization, and immediate good control.

Initial laboratory examination showed a white blood cell (WBC) count of 16000 (Ul), Lactic Acid of 2.33 (mmol/L), Alanine transaminase (ALT) of 767 (U/L), and serum glucose of 8.29 (mmol/L). The focused assessment sonography for trauma (FAST) was found positive, whereas the chest and pelvic X-rays and head CT were normal. Chest CT revealed bilateral lung contusion with minimal right sided pneumothorax and fracture of first and fifth ribs. Abdominal CT scan revealed large liver contusion and laceration (Grade 4) with blush and moderate free haemoperitonium (3 quadrants) (Figure 1). Patient was managed nonoperatively with immediate shift for interventional radiology to attempt angioembolization of the bleeding vessels. Two anomalies in hepatic arteries origin including left hepatic artery originated from the celiac trunk and right hepatic artery from the superior mesenteric artery were reported. Both vessels were selectively cannulated and bilateral gel foam embolization of the bleeders was achieved with immediate satisfactory results (Figure 2).

**Figure 3:**
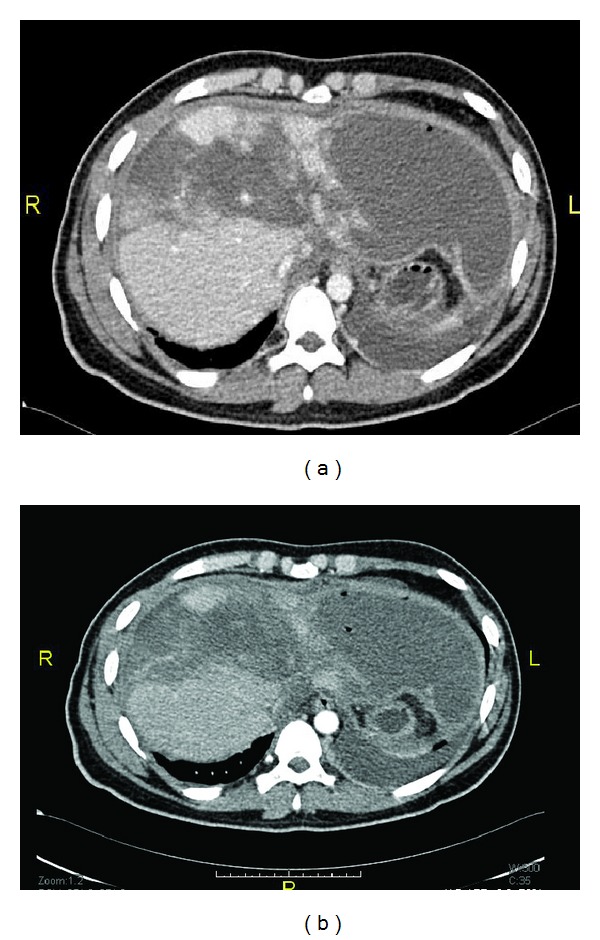
Follow-up abdomen CT (after TAE) showed massive liver necrosis with sparing of the lower part of the right lobe.

A follow-up abdomen CT (48 hrs) revealed left sided pleural effusion and massive liver necrosis (MLN) with significant increase of peritoneal fluid along the left paracolic and pelvic compartments (Figure 3).

**Figure 4:**
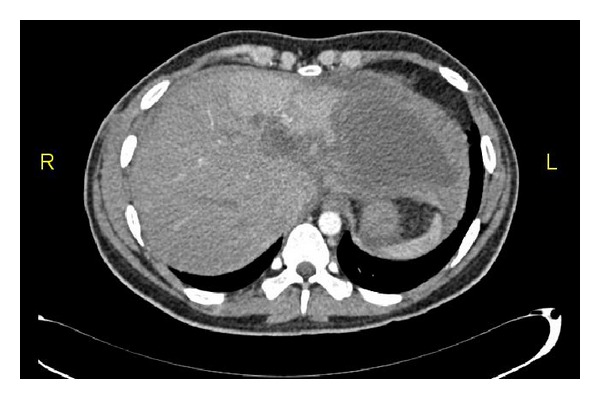
Follow-up abdomen CT (after 4 weeks of the TAE) showed start of liver regeneration and resolution of left lobe cystic changes.

**Figure 5:**
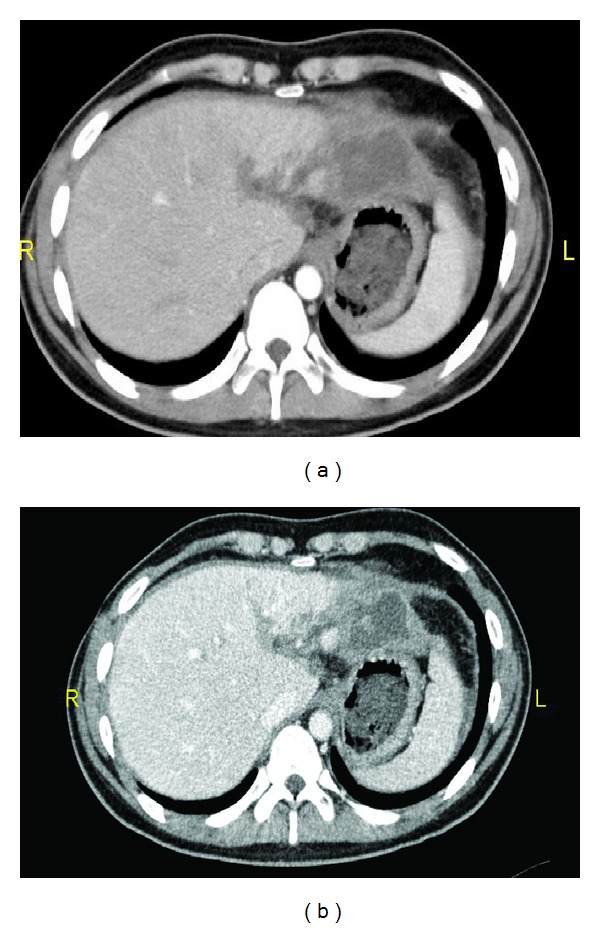
Follow-up abdomen CT (one year): (a) regeneration of right lobe and (b) shrunken left lobe.

Another CT after 4 weeks of TAE revealed that both lobes of the liver had diffuse nonenhancing and nonhomogenous areas replacing almost the left lobe and segments V and VIII of the right lobe with fluid loculation but no free fluid (Figure 4). Outpatient follow-up CT scan (12 months later) showed progressive resolution of necrotic areas with fluid replacement and remarkable regeneration of liver tissue (Figure 5). In particular, the right lobe regenerated quickly literally replacing the left lobe which remained atrophic. Patient was doing well with regular outpatient follow-up.

## 3. Discussion

The management of liver injury is challenging particularly for severe grades (Grades III–V) [14]. Though recent advances in resuscitation and adoption of new diagnostic and therapeutic approaches have reduced the mortality, the rate of associated morbidities remains significant [7]. An earlier study demonstrated that hepatic angiography is an ideal option to control bleeding and delayed complications in high risk patients who presented with high-grade liver injury associated with arterial or hepatic venous injuries [15]. Angiography and subsequent embolization (angioembolization) are more useful for the management of liver injury particularly that with CT scan blush [16]. It has been reported that angioembolization is a potential tool for controlling hepatic bleeding with high efficacy rate [17]. Although angioembolization is an effective modality, it still represents higher morbidity due to renal impairment, localized hepatic necrosis, abscess, necrosis of biliary tract, and cholecystitis [16, 18]. New myriad of complications were reported with the increased and successful utilization of nonoperative management strategies and frequent use of transarterial embolization for liver injuries with bleeding [14, 19–23].

Major liver devascularization (MLN) is a very peculiar entity of great interest. It is related to the disruption of vascular inflow to a liver portion either by the original trauma or following angioembolization [8]. MLN has a range of clinical presentation according to its extent and the patient response that ranges from laboratory derangement to clinical manifestation (i.e., abdominal pain, feeding intolerance, and bile leaks). Moreover, contrast-enhanced abdominal CT confirms an even more complicated course related to the development of sepsis and multiorgan dysfunction [24]. It has been observed that these complications are associated with higher incidence of worse outcomes. Several studies suggested that patients with significant necrosis should undergo hepatic resection before the onset of complications [25, 26]. In addition, surgical debridement is also challenging and carries significant risk of morbidity. Debate is ongoing about the indications for TAE, predictors of severe complications, and the best management approach for MLN. The majority of studies that follow initial damage control operative intervention and postoperative TAE would recommend early major debridement of subsequent necrosis [18]. However, little is known about the implications of TAE for patients who are treated nonoperatively [7, 12, 18, 27].

To the best of our knowledge, there are only few reported cases of MLN complicating TAE which are treated nonoperatively. The current report represents a case of severe liver injury (Grade IV with blush) treated with nonoperative management using TAE and complicated by MLN. The patient developed major liver devascularization to some extent related to his vascular anomalies with TAE being less selective. The pattern of liver enzymes, WBC count, clinical presentation, and subsequent scanning confirmed the diagnosis. Nevertheless, the patient managed to tolerate liver devascularization and he showed a smooth recovery without developing severe complications such as hepatic failure, sepsis, abdominal compartment, late bleeding, or multiorgan derangements. Follow-up CT scans demonstrated a great regeneration and full function, recovered liver.

In conclusion, we assume that patients with high-grade severe liver injuries could be managed carefully according to a clear protocol. Special attention should be given to the major associated morbidities. A selective or rather tailored multidisciplinary approach to manage the morbid complications would represent the best recommended strategy for success and better outcomes in severe liver injury patients. Further studies are needed to support our assumption.

## References

[1] NCIPC: Web-based Injury Statistics Query and Reporting System (WISQARS). http://www.cdc.gov/injury/#.

[2] Bonatti H, Calland JF (2008). Trauma. *Emergency Medicine Clinics of North America*.

[3] Clancy TV, Maxwell JG, Covington DL, Brinker CC, Blackman D (2001). A statewide analysis of level I and II trauma centers for patients with major injuries. *Journal of Trauma*.

[4] Matthes G, Stengel D, Seifert J, Rademacher G, Mutze S, Ekkernkamp A (2003). Blunt liver injuries in polytrauma: results from a cohort study with the regular use of whole-body helical computed tomography. *World Journal of Surgery*.

[5] Gourgiotis S, Vougas V, Germanos S (2007). Operative and nonoperative management of blunt hepatic trauma in adults: a single-center report. *Journal of Hepato-Biliary-Pancreatic Surgery*.

[6] Asensio JA, Roldán G, Petrone P (2003). Operative management and outcomes in 103 AAST-OIS grades IV and V complex hepatic injuries: trauma surgeons still need to operate, but angioembolization helps. *Journal of Trauma*.

[7] Dabbs DN, Stein DM, Scalea TM (2009). Major hepatic necrosis: a common complication after angioembolization for treatment of high-grade liver injuries. *The Journal of trauma*.

[8] Di Saverio S, Moore EE, Tugnoli G (2012). Non operative management of liver and spleen traumatic injuries: a giant with clay feet. *World Journal of Emergency Surgery*.

[9] Pachter HL, Hofstetter SR (1995). The current status of nonoperative management of adult blunt hepatic injuries. *American Journal of Surgery*.

[10] Zago TM, Pereira BMT, Calderan TRA, Godinho M, Nascimento B, Fraga GP (2012). Nonoperative management for patients with grade IV blunt hepatic trauma. *World Journal of Emergency Surgery*.

[11] Hagiwara A, Yukioka T, Ohta S (1997). Nonsurgical management of patients with blunt hepatic injury: efficacy of transcatheter arterial embolization. *American Journal of Roentgenology*.

[12] Ciraulo DL, Luk S, Palter M (1998). Selective hepatic arterial embolization of grade IV and V blunt hepatic injuries: an extension of resuscitation in the nonoperative management of traumatic hepatic injuries. *Journal of Trauma*.

[13] Lin B-C, Wong Y-C, Lim K-E, Fang J-F, Hsu Y-P, Kang S-C (2010). Management of ongoing arterial haemorrhage after damage control laparotomy: optimal timing and efficacy of transarterial embolisation. *Injury*.

[14] Kozar RA, Moore FA, Cothren CC (2006). Risk factors for hepatic morbidity following nonoperative management: multicenter study. *Archives of Surgery*.

[15] Poletti PÅ, Mirvis SE, Shanmuganathan K, Killeen KL, Coldwell D (2000). CT criteria for management of blunt liver trauma: correlation with angiographic and surgical findings. *Radiology*.

[16] Misselbeck TS, Teicher EJ, Cipolle MD (2009). Hepatic angioembolization in trauma patients: indications and complications. *Journal of Trauma*.

[17] Ahmed N, Vernick JJ (2011). Management of liver trauma in adults. *Journal of Emergencies, Trauma and Shock*.

[18] Mohr AM, Lavery RF, Barone A (2003). Angiographic embolization for liver injuries: low mortality, high morbidity. *Journal of Trauma*.

[19] Duane TM, Como JJ, Bochicchio GV, Scalea TM (2004). Reevaluating the management and outcomes of severe blunt liver injury. *Journal of Trauma*.

[20] Christmas AB, Wilson AK, Manning B (2005). Selective management of blunt hepatic injuries including nonoperative management is a safe and effective strategy. *Surgery*.

[21] Monnin V, Sengel C, Thony F (2008). Place of arterial embolization in severe blunt hepatic trauma: a multidisciplinary approach. *CardioVascular and Interventional Radiology*.

[22] Carrillo EH, Spain DA, Wohltmann CD (1999). Interventional techniques are useful adjuncts in nonoperative management of hepatic injuries. *Journal of Trauma*.

[23] Goldman R, Zilkoski M, Mullins R, Mayberry J, Deveney C, Trunkey D (2003). Delayed celiotomy for the treatment of bile leak, compartment syndrome, and other hazards of nonoperative management of blunt liver injury. *American Journal of Surgery*.

[24] Anderson IB, Al Saghier M, Kneteman NM, Bigam DL (2004). Liver trauma: management of devascularization injuries. *Journal of Trauma*.

[25] Strong RW, Lynch SV, Wall DR, Liu C-L (1998). Anatomic resection for severe liver trauma. *Surgery*.

[26] Smadja C, Traynor O, Blumgart LH (1982). Delayed hepatic resection for major liver injury. *British Journal of Surgery*.

[27] Gaarder C, Naess PA, Eken T (2007). Liver injuries-Improved results with a formal protocol including angiography. *Injury*.

